# SipNose-topiramate: a potential novel approach to binge eating management

**DOI:** 10.1186/s40337-023-00825-9

**Published:** 2023-06-26

**Authors:** Ayala Kobo-Greenhut, Adit Zohar-Beja, Liron Hadar, Lior Itzhaki, Avraham Karasik, Yoseph Caraco, Hilel Frankenthal, Daniel Shahaf, Dana Ekstein, Iris Shichor, Eitan Gur

**Affiliations:** 1 Risk Management, Validation, Regulation, Haifa, Israel; 2 Eating Disorder Department, Sheba Medical Center, Tel HaShomer, Israel; 3 SipNose Ltd., Yokneam, Israel; 4 Clinical Pharmacology Unit, Division of Medicine, Hadassah-Hebrew University Medical Center, Jerusalem, Israel; 5 Ziv Medical Center, Safed, Israel; 6 Zefat Academic College, Safed, Israel; 7 Department of Neurology and Agnes Ginges Center for Neurogenetics, Hadassah Medical Center, Jerusalem, Israel; 8 Faculty of Medicine, The Hebrew University of Jerusalem, Jerusalem, Israel

**Keywords:** Binge eating disorder, SipNose-topiramate, Direct nose-to-brain

## Abstract

**Background:**

Binge-eating disorder) BED) is the most common eating disorder in the United-States. Daily, orally administered topiramate has shown BED treatment efficacy, with two major limitations: frequent and severe side effects and slow time-to-effect. SipNose is a novel non-invasive intranasal direct nose-to-brain drug delivery platform that delivers drugs to the central nervous system consistently and rapidly. Herein, we study a SipNose-topiramate combination product, as an acute “as needed” (PRN) solution for BED management.

**Methods:**

First, SipNose-topiramate’s pharmacokinetics (PK) and safety was evaluated. The second part aimed to demonstrate its PRN-treatment feasibility in terms of usability and potential efficacy in reducing the number of binge-eating events. Twelve BED patients were studied over three time periods; 2-weeks of baseline monitoring [BL], 8-weeks of treatment [TX], and 2-weeks of follow up [FU].

**Results:**

The PK profile showed peak plasma levels at 90 min post-administration, a t_1/2_ > 24 h and consistent topiramate delivery with no adverse events. In the second part, 251 treatments were self-administered by the patient participants. There was a significant reduction from baseline to treatment periods in mean weekly binge-eating events and binge-eating event days per week. This was maintained during the follow up period. Efficacy was corroborated by improved patient illness severity scales. There were no adverse events associated with any administered treatments. Patients were exposed to less drug when compared with accepted oral dosing.

**Conclusions:**

This study introduces a SipNose-topiramate drug-device combination as a potentially safe, effective, and controlled method for BED management. Its findings introduce a potential approach to BED management both as an intranasal and as a PRN therapy for reducing binge-eating events, with a large-scale reduction in patient drug exposure and side effects and with improved patient quality of life. Further studies are needed with larger patient populations to establish SipNose-topiramate as a mainstream treatment for BED.

*Trial registration*: Registration number and date of registration of the clinical studies reported in this article are as follows: 0157-18-HMO, August 15th 2018 and 6814-20-SMC, December 2nd 2020.

**Supplementary Information:**

The online version contains supplementary material available at 10.1186/s40337-023-00825-9.

## Abstract (plain language summary)

Binge eating disorder (BED) is a common eating disorder. Daily oral topiramate treatment has shown efficacy in clinical studies and off-label use, with frequent and severe side effects. SipNose is a novel, rapid and consistent direct nose-to-brain drug delivery platform. This study evaluates a SipNose-topiramate combination product, as an innovative acute “as needed” (PRN) BED treatment solution. SipNose-topiramate's pharmacokinetics (PK) and safety demonstrated consistent, dose-dependent topiramate delivery with no adverse events. SipNose-topiramate was studied vis-à-vis its safety and feasibility as a PRN-treatment for reducing the number of binge-eating events. 12 BED patients were studied (2-weeks baseline monitoring, 8-weeks treatment, 2-weeks follow-up). Patients were instructed to self-administer the drug when they feel an urge to binge-eat. Two hundred fifty-one treatments were administered. When compared with daily oral dosing, lower doses were used with no adverse events and minimal side effects. Baseline to treatment periods showed significant reduction in mean weekly binge-eating events and binge-eating event days-per-week. This was maintained during follow-up. Improved illness severity scales corroborated the improved feasibility outcomes. In conclusion, this study introduces SipNose-topiramate as a potential “as needed” intranasal treatment for BED that is safe, effective, and reduces drug exposure and side effects. Additional studies are needed to validate SipNose-topiramate as a BED management therapy.

**Supplementary Information:**

The online version contains supplementary material available at 10.1186/s40337-023-00825-9.

## Background

Binge eating disorder (BED) is defined in the American Psychiatric Association (APA) diagnostic and statistical manual of mental disorders (DSM-5) as “recurrent uncontrolled and distressing episodes of binge eating. An episode of binge eating, is characterized by eating, in a discrete period of time, an amount of food larger than most people would eat in a similar period, under similar circumstances, and with a sense of lack of control over eating during the episode [1].” Most binge-eating episodes (binge-eating events) develop following an overwhelming urge to binge-eat [1, 2].

The prevalence of BED varies widely between studies and counties, ranging from 0.3 to 3.6%. In the United States, the lifetime BED prevalence in the general population is conservatively estimated to be 0.85–2.6% [2], making it the most common eating disorder in the US, more common than anorexia nervosa and bulimia nervosa combined [3–5]. The World Mental Health Survey population-based estimates of BED prevalence among adults in different countries varied widely across settings [3]. In a meta-analysis of studies completed before the year 2018, past-year prevalence of DSM-5 BED in adults overall was estimated to be 1.3% (95% CI 0.6–2.3%): 0.3% (95% CI 0.1–0.6%) for men and 1.5% (95% CI 1.2–1.7%) for women. Subsequent methodologically rigorous population-based studies provide widely varying estimates, though BED is frequently underdiagnosed [6–9].

Current treatment options for BED include both psychotherapy and pharmacotherapy [10]. Recent American Psychiatric Association guidelines for BED treatment recommend “that adults with binge-eating disorder who prefer medication or haven’t responded to psychotherapy alone be treated with either an antidepressant medication or lis-dexamfetamine [11].” To date, lisdexamfetamine, is the only FDA approved medication for treating BED [12]. Despite its reported efficacy, patients treated with lisdexamfetamine are exposed to variety of unwanted side effects including the major risk of abuse that may lead to dependence. Antidepressants have shown some promise [13] though most studies have shown modest results with only a reduction in binge-eating, but without clinically significant weight loss, or sustained benefit [14–18]. In a recent study, dasotraline, a dopamine and norepinephrine reuptake inhibitor (DNRI) demonstrated benefit in reducing binge-eating behavior [19, 20]. However, its development program was discontinued, due to regulatory hurdles [21].

Topiramate, an anti-seizure medication, has demonstrated clinical efficacy in treating BED [4, 22]. In several clinical studies assessing weight loss topiramate decreased the frequency of binge-eating events [22–24], though its tolerability profile can limit its use. Neurredine recently published a meta-analysis of topiramate use in BED [25]. In the three studies found eligible for analysis, topiramate was found to be significantly more efficacious than placebo in reducing the number of binge-eating events per week, the number of binge-eating days per week, and patient’s weight. However, participants in the topiramate groups withdrew more frequently than placebo participants due to safety reasons. Topiramate use has therefore been limited primarily due to negative neurological and cognitive side effects [4, 22]. Thus, there remains a great medical need for better tolerated BED treatments.

Several lines of evidence support topiramate's potential as an effective BED therapy. Firstly, three randomized control trials have demonstrated topiramate's treatment efficacy in individuals with BED [22–25]. Secondly, topiramate has shown efficacy in reducing binge-eating behavior in individuals with bulimia nervosa [26]. Lastly, topiramate has been approved by the FDA for long-term weight loss treatment when used in combination with phentermine [27, 28]. In all the above examples, oral administration was used as a daily treatment.

Currently, marketed dosages of topiramate are based on daily oral capsule/tablet administration. Oral topiramate delivery, though effective, has two major limitations. Firstly, high systemic levels, and in turn, high systemic dosing are needed in order to achieve therapeutic topiramate concentrations in the brain. Unfortunately, daily oral topiramate administration is associated with frequent and severe side effects, including paresthesias, speech disorders, fatigue, dizziness, somnolence, nervousness, psychomotor slowing, abnormal vision and fever [29]. These dose dependent and reversible adverse events/side effects occur in more than 10% of patients, and preclude many patients from its continued use [29]. Furthermore, the required high systemic doses increase side effect frequency and severity. Additionally, oral administration results in slow drug delivery to the brain and delayed time to affect. This is a limitation of any orally administered medications, including lisdexamfetamine and antidepressants recommended in the recent American Psychiatric Association guidelines [11, 30]. To date, there is no practical market-ready product that can offer an alternative drug delivery method to overcome those limitations.

**Fig. 1:**
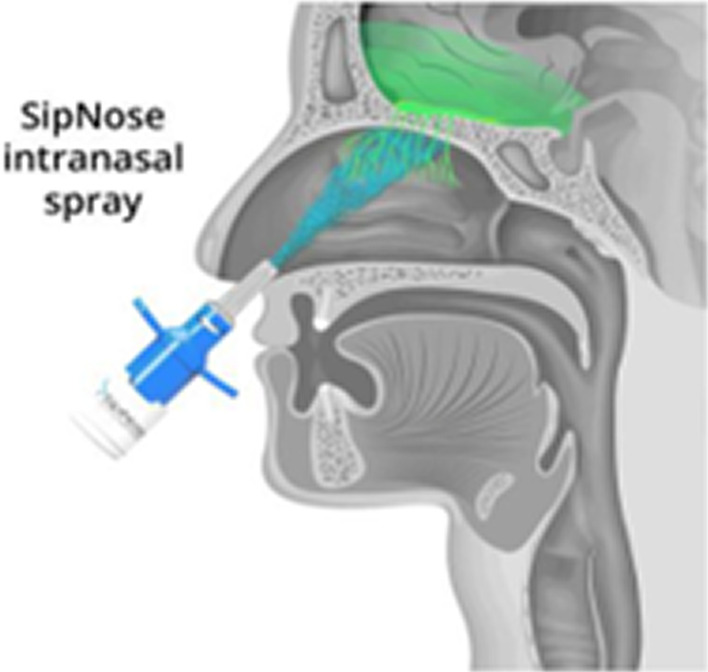
SipNose device and Clinical evidence for SipNose superior delivery to the olfactory epithelium (see Additional file 1: Fig. S1, for further evidence of SipNose’s drug delivery to the olfactory epithelium)

Intranasal (IN) delivery is a drug delivery route utilized in various therapeutic areas including CNS therapies [31, 32]. All currently commercially available intranasal drug-delivery devices are intended for local or systemic distribution and deliver aerosolized drug via nasal mucosa-to-bloodstream absorption. Although nasal-to-systemic delivery has its advantages, such as avoiding the hepatic first-pass effect encountered in the oral route, the drug needs to reach high systemic levels in order to cross the blood-brain-barrier and achieve steady therapeutic brain tissue concentrations. As such, nasal-to-systemic drug delivery has some of the limitations of the oral route. However, direct nose-to-brain drug delivery technology allows for rapid non-invasive delivery of small and large molecules to the CNS. It therefore has the potential for CNS drug delivery without the limitations of other drug delivery routes. In fact, due to its direct CNS tissue delivery, lower doses are needed when compared with systemic delivery [33, 34]. Furthermore, in a recent study, Kobo-Greenhut, et al. highlight the qualitative clinical advantages of intranasal direct nose-to-brain delivery over invasive intrathecal and intracerebroventricular CNS drug delivery [35]. SipNose is one such non-invasive direct nose-to-brain (DNTB) drug delivery platform [35] (see Fig. 1). The SipNose DNTB technology takes advantage of the nasal cavity's physiological structure and its proximity to the olfactory and trigeminal nerve pathways, to allow for efficient drug absorption and delivery from the upper nasal cavity to the CNS along these neuronal pathways, thereby bypassing the BBB [36, 37].

This study introduces a novel drug-device combination product that utilizes the SipNose non-invasive DNTB platform to deliver topiramate to the CNS. This drug-device combination product aims to provide an effective acute “as needed” (PRN) treatment for stopping the progression of an eating urge to a binge-eating event, and a potential solution for the limitations of daily topiramate administration via systemic drug delivery methods. The physiologic rationale underlying this drug-device combination treatment takes advantage of SipNose's ability to rapidly deliver the topiramate from the upper nasal cavity directly to the brain. These two properties of rapid and direct brain delivery are anticipated to provide acute treatment to reduce/stop a binge-eating event from developing at its early stages. Furthermore, PRN direct nose to brain treatment could, ostensibly, reduce the required topiramate dose, and eliminate daily usage steady state drug exposure, thereby diminishing adverse event frequency and severity.

Preclinical studies of the topiramate-SipNose product have successfully demonstrated preliminary product safety in delivering high doses of intranasal topiramate, a correlation between brain and plasma topiramate levels following IN administration, and safety and pharmacokinetic (PK) data for repeated IN dosing. The current study is designed to provide a preliminarily evaluation of this drug-device combination as potential BED treatment that is novel in its acute “as needed” treatment approach and its intranasal drug delivery method. It also aims to provide preliminary data regarding its potential to diminish the required topiramate dose, frequency and adverse event frequency/severity.

The study is designed in two parts. The first aims to preliminarily evaluate the SipNose-topiramate combination product's safety and pharmacokinetics (PK). The second part aims to provide preliminary proof-of-concept efficacy and feasibility data as a PRN treatment for reducing binge-eating events in BED patients.

## Method

### Study design

Both study parts were designed to be a single center, open label study, established by trained study staff.

### Part I—pharmacokinetics (PK)

*Setting—*Part I took place in the Hadassah Medical Center, Jerusalem, Israel, during the period of September 2018–March 2019.

*Participant recruitment—*The site advertised the study based on advertisement approved by the local IRB (Tofes 10).

*Participants—*Part I participants included 8 healthy volunteers in total. Each subject participated in 3 study cohorts. Part I inclusion and exclusion criteria are as follows:

Inclusion criteria.

- Age 18–45 years.
- Body mass index: 22–27 kg/m^2^.
- Healthy.
- Written informed consent.

Exclusion criteria.

- Age below 18 years.
- Women who were pregnant or breast-feeding.
- Women of childbearing potential could be enrolled if highly effective non hormonal contraception was used.
- Subjects treated by any drug on a regular basis during the study.
- Smoking on a routine basis.
- Nose trauma in the preceding 10 years.
- Allergic rhinitis.
- Any nasal congestion or physical blockage of either nostril, or deviated nasal septum as determined by nasal examination.
- History of kidney stones or urinary tract disease.
- Medically relevant safety laboratory result deviations during the screening evaluation, which could pose a safety risk to the subject, if included in the study. The safety risk was determined by study investigators.

*Design—*Part I studied the drug’s PK/safety profile.

Enrolled subjects were officially recruited only after laboratory evaluation results were received. In the event of abnormal initial laboratory results, the investigators were able to repeat the test once to confirm eligibility.

This part was designed to only provide a preliminary assessment of the SipNose-topiramate device's PK and safety profile. As such, the study was not expected to show statistical significance or statistical power and no formal sample size calculation was performed. The planned sample size was deemed adequate by the sponsor and investigators for this purpose. Study staff were responsible for study procedures, including drug administrations and blood sampling.

Drug safety was evaluated based on routine laboratory evaluations (i.e., biochemistry, complete blood count, coagulation studies, blood pH and urine analysis), adverse events and side effects. Adverse events were defined as unexpected medical reactions [38, 39]. PK evaluation was derived from plasma topiramate concentrations in samples obtained prior to and 10, 30, 60, 90, 120, 180, 360, 540 min and 24 h following topiramate administration.

Topiramate half-life is known to be approximately 21 h [40], thus study investigators anticipated a high likelihood of measurable topiramate plasma concentrations at 24 h post administration but low likelihood after 48 h post-administrations. Participants in Part I were studied in three separate time periods as 3 study cohorts. The time interval between successive cohorts was at least 7 days. A null drug level at Time 0 for Cohort #2 and #3 demonstrated complete drug elimination between subsequent cohorts.

- Cohort #1: Participants were administered 30 mg IN topiramate, 15 mg in each nostril.
- Cohort #2: Participants were administered 60 mg IN topiramate, 30 mg in each nostril.
- Cohort #3: Participants were administered repeated doses. The size of each dose, total number of doses per day and time interval between consecutive doses was set by the Safety Monitoring Committee based on the safety and pharmacokinetic data obtained from Cohorts 1 and 2 (or PK data from Cohort 2 only).

The repeated doses were as follows:

Dose 1: 60 mg (30 mg in each nostril)

Dose 2: 60 mg (30 mg in each nostril), 60 min following dose 1 (cumulative dose 120 mg)

Dose 3: 60 mg (30 mg in each nostril) 300 min following dose 2 (cumulative dose 180 mg)

#### Variables

- Adverse events and side effects were evaluated based on safety monitoring, physical examination, blood biochemistry, complete blood count, coagulation tests, blood pH, urine analysis, pregnancy declaration/test and electrocardiogram.
- Pharmacokinetics were evaluated, including dose response relationship between cohorts #1 and #2; and cumulative dose response in cohort #3.

### Part II—a “proof-of-concept” study

*Setting*—Part II was performed via the Department of Eating Disorders at Sheba Medical Center, Tel HaShomer, Israel, between December 2020 and November 2021. This was a 12-week open label study in which BED patients self-administered the treatment at home on an “as needed” basis.

*Patient recruitment*—Patients with moderate-severe Binge Eating Disorder (BED) were recruited from outpatient clinicians, the unit at which the study was conducted and via radio, newspaper, and advertisement approved by the local IRB. Subjects were trained to use the SipNose device for intra-nasal topiramate delivery. Subjects who did not satisfy training criteria after SipNose device training could not be included in the next study phase.

The study staff were responsible for study procedures.

*Participants*—Fourteen patients with BED were enrolled in the study’s second part. All had met DSM-5 criteria for moderate-severe BED for 6 months, at minimum, prior to study enrollment. Twelve patients participated for the entire study duration. Two patients left the study for personal reasons, unrelated to study participation.

Part II inclusion and exclusion criteria are as follows:

Inclusion criteria.

- Part II participants were included if they met diagnostic criteria for BED as determined by the Structured Clinical Interview for DSM-5 (SCID) [41] and supported by the Eating Disorder Examination (EDE) [42].

Exclusion criteria.

- Body mass index < 18 kg/m^2^ or > 45 kg/m^2^.
- Patients who had active disease involving the nasal sinuses or history of chronic sinusitis, rhinorrhea, or nasal congestion.
- Women who were pregnant or lactating and women of childbearing potential who aren’t taking adequate contraceptive measures. All women of childbearing potential were required to have a negative pregnancy test before entering the study.
- Patients who had a lifetime history of a DSM-5 diagnosis of a substance abuse or dependence disorder, except for nicotine abuse or dependence (as determined by psychiatric history, SCID interview, and urine toxicology; see below).
- Patients who had a lifetime history of a DSM-5 diagnosis of psychotic disorder, bipolar disorder, or dementia.
- Patients who had a history of a personality disorder (e.g., schizotypal, borderline, or antisocial) which might interfere with assessment or compliance with study procedures.
- Clinically unstable medical disease, including cardiovascular, hepatic, renal, gastrointestinal, pulmonary, metabolic, endocrine, or any other systemic disease that could interfere with BED diagnosis, assessment, or treatment with topiramate.
- Patients who required treatment with any drug that could adversely interact with or obscure SipNose-topiramate activity
- Patients who had received an experimental drug or used an experimental device within 30 days.
- Deviations in medically relevant safety laboratory results obtained during the screening evaluation that could pose a safety risk to the patient if included in the study. Safety risk was determined by study investigators.
- Any nasal congestion or physical blockage in either nostril, or deviated nasal septum as determined by nasal examination.
- History of significant cardiovascular, hepatic, renal, hematologic, gastrointestinal, endocrine, immunologic, dermatologic, neurologic, or psychiatric disease that could potentially impact the safety of the patient or metabolism of the drug.
- Patients who suffered from acute or chronic pulmonary disease.

*Design*—This phase was designed to compare patient disease severity and treatment effect between baseline, treatment, and follow-up time periods (phases). Enrolled patients participated in all three phases.

*Enrollment assessment* Study patients underwent a screening visit during which the research team collected baseline data and evaluated the patients’ suitability for study enrollment according to inclusion criteria. Visit content can be seen in Table 1.

**Table 1:** Part II patient visit content

Visit # and time	Content
Visit 1 time = 0	*Screening* Signing consent form (ICF) Confirming eligibility for study participation Detailed review of past medical history Structured Clinical Interview (SCID) [41] Eating Disorder Examination (EDE) [42] Review of concomitant medications and general health status in the week preceding the visit Demographics, including height Weight measurement Complete physical examination, including nasal mucosa examination Drawing safety screening laboratory blood and urine samples Urine pregnancy test for women t0 baseline: Clinician Global Impression (CGI-S) [52] and Yale-Brown Obsessive Compulsive Binge Eating Scale (Y-BOCS-BE) [44, 52]
	*Diaries* Training on diary documentation during the baseline period Electronic diary system (e-Diary) training, including the two diary forms (Diary #1 and Diary #2) patients were requested to fill during the study Receiving e-Diary IFU, a user name and password Patients were requested to complete the diaries during the subsequent two weeks, aimed to characterize their binge-eating behavior (Diary #1)
Visit 2 BP 2nd week Time = 2 week	Baseline period–assessment t2 - CGI-S t2 - Y-BOCS-BE Number of binge-eating days per week Number of binge-eating events per week
	Decision to include/exclude. Criteria to continue including the patient in the study Completed diaries (visit 2—have to show at least 2 binge-eating days per week) Laboratory baseline that shows no deviation from required value
	Guide for included patients Receiving treatment kits to cover the maximum allowed treatments per one week which are sufficient for use prior to the next visit Receiving a quick reminder of how the product shall be used, and how to fill the diaries
Visit 3: TP 1st week Time = 3 week	Treatment period—assessment 1 Review of possible adverse events Return the devices from the 1st treatment week Review the e-diaries of the 1st treatment week and receive inputs which could potentially improve completion of the e-diaries and data collection t3 - CGI-S and Y-BOCS-BE scores Receive devices for the next two (2) treatment weeks
Visit 4: TP 2nd week Time = 4 week	Review of possible adverse events Review e-Diaries with the study staff and receive inputs t4 - CGI-S and Y-BOCS-BE scores
Visit 5: TP 3rd week Time = 5 week	Review of possible adverse events t5 - CGI-S and Y-BOCS-BE scores Return the devices of the preceding two (2) treatment weeks Receive devices for the next two (2) treatment weeks
Visit 6: TP 4th week Time = 6 week	Review of possible adverse events t6 - CGI-S and Y-BOCS-BE scores
Visit 7: TP 5th week Time = 7 week	Review of possible adverse events t7 - CGI-S and Y-BOCS-BE scores Return the devices of the preceding two (2) treatment weeks Receive devices for the subsequent three (3) treatment weeks
Visit 8 TP 6th week Time = 8 week	Review of possible adverse events t8 - CGI-S and Y-BOCS-BE scores
Visit 9: TP 7th week Time = 9 week	Review of possible adverse events t9 - CGI-S and Y-BOCS-BE scores
Visit 10: 8th weekend of TP Time = 10 week	Review of possible adverse events Physical examination Drawing of laboratory blood samples for safety testing Return the devices of the preceding three (3) treatment weeks t10 - CGI-S and Y-BOCS-BE scores Weight measurement

BP baseline phase, TP treatment phase, CGI-S clinician global impression, YBOCS-BE Yale-Brown obsessive compulsive binge eating scale

#### Study phases

- *Baseline phase* This phase lasted 2 weeks, during which data was collected about each patient’s baseline binge-eating behavior characteristics. Patients were requested to maintain diaries and report on each urge to binge-eat and/or binge-eating event they experienced. The urge to binge-eat was self-reported as a feeling of uncontrollable urge to binge-eat. Although urges to binge-eat aren’t an established measure in eating disorders, they are inherent to, and precede many binge-eating events in BED patients. Study investigators hypothesized that the urge to binge-eat was the logical and preferred time point for any PRN treatment that aims to reduce binge-eating frequency. Binge-eating episodes/events were defined in accordance with DSM-5 criteria [1] as rapid, uncontrollable eating of unusually large food quantities. Enrolled patients had a known BED diagnosis and could therefore self-identify (at times during, at times after) a binge-eating event during which they ate large quantities rapidly and uncontrollably. As such, food quantity and eating timeframe were not delineated. Rather, patients were instructed to self-identify a binge-eating event based on prior BED binge-eating experience.
- *Treatment phase* This phase lasted 8 weeks, during which patients self-treated with the SipNose-topiramate product. Patients were instructed to take an intranasal topiramate dose, whenever they felt an urge to binge-eat or in the early stages of binge-eating events. In the latter, patients were instructed to self-treat regardless of whether the binge-eating began without sensing a preceding urge to binge-eat, or whether they felt the urge to binge-eat but did not self-treat at the time. Patients self-administered a 60 mg topiramate dose. After 10 min, if they felt the first dose was ineffective, patients self-administered a second single 60 mg dose (total dose 120 mg). After 10 min subjects could administer a third 60 mg doses (total dose 180 mg) if they felt that the first two doses were ineffective. Patients were instructed to consider a dose ineffective if they perceived an ongoing loss of control over the urge to binge-eat or an inability to stop eating once a binge-eating event had begun. Patients were requested to maintain daily diaries documenting their urges to binge-eat, their binge-eating events, which urge to bing-eat/binge-eating events were treated, doses administered, and whether the treatment was helpful. The treatment phase included follow up visits and an end-of-treatment visit. Visit content can be seen in Table 1.
- *Follow-up phase* This phase lasted 2-weeks, was treatment-free and involved data collection via patient follow-up visits regarding binge-eating behavior; urges to binge-eat and binge-eating events.

Data was also collected in search of late-onset and negative treatment withdrawal effects.

Treatment effect was assessed based on the change in variables when comparing the before (Baseline), during (Treatment) and after (Follow-up) treatment phases.

Initially, all visits were planned as in-person visits performed in the clinic. As a result of the Covid-19 pandemic restrictions, this was modified to only two in-person in-clinic visits while the others were conducted either in-person at the patient's home or via remote video.

At study commencement and conclusion, every patient in Part II underwent a psychological and physical physician examination and laboratory testing.

During the three study phases, patients filled daily electronic diaries, and participated in weekly meetings, in which control reports were filled by a clinical dietitian.

Study staff reviewed the e-diary information for each study subject and were responsible for evaluating whether the definition of “binge-eating events” was met as defined by study protocol and whether the dosing documentation was completed appropriately. Discrepancies in the diary were resolved by study staff and reflected in the case report forms.

#### Variables and outcomes

Binge-eating events were selected as the primary variable reflecting BED severity. As such, the study’s primary outcomes for Part II, were to demonstrate the SipNose-topiramate product’s preliminary effect on binge-eating event frequency, BED illness severity, and product tolerance in terms of adverse events and side effects. The secondary outcomes include treatment effect on urge to binge-eat episodes frequency and preliminary data on possible lingering “tail” treatment effects as determined by Follow-up Phase data.

The following variables were evaluated in Part II.

Outcomes: treatment effect on binge-eating events/urge to binge-eat frequency:

- Number of binge-eating events.
- Number of urge to binge-eat events.
- Number of days in which binge-eating events and urge to binge-eat events occurred.
- Control variables for urge to binge-eat/binge-eating events frequency included the weekly number of urge to binge-eat events, and the number of days in which an urge to binge-eat event occurred in the Baseline Phase.

Outcomes: BED illness severity:

- In addition to binge-eating event frequency, control variables for BED illness severity included patient severity of illness scoring and post-treatment condition scoring. These were evaluated through two clinical scales [43, 44]:
- The Clinical Global Impression—Severity (CGI-S) [43] is a 7-point scale that is used by the clinician to describe patient severity of illness. It is a general scale used for a variety of psychiatric conditions and is not specific to BED. The scale ranges from 1 = normal, 2 = borderline ill, 3 = mildly ill, 4 = moderately ill, 5 = markedly ill, 6 = severely ill and 7 = among the most extremely ill patients.
- The Yale-Brown obsessive compulsive Scale modified for the assessment of binge eating (YBOCS-BE) [44] is a clinician-rated BED specific scale that measures the degree of obsessive and compulsive binge-eating behaviors. Total scores range from 0 to 40. A score of 0–7 is sub-clinical, 8–15 is mild, 16–23 is moderate, 24–31 is severe and 32–40 is extreme. High YBOCS-BE total and subscale scores represent greater severity of illness.

Outcomes: adverse events and side effects:

- The following were obtained for safety monitoring: physical examination (including an ear, nose, and throat exam); vital signs (blood pressure, pulse rate, and temperature); blood laboratory tests (biochemistry and CBC), and urine toxicology (cannabinoid, benzodiazepine, amphetamine, and methadone metabolite screening).

### Statistical analysis

Statistical analysis of data obtained from the diaries, was established using SAS Version 9.4. Changes in variables, including the number and proportion of binge-eating events and urge to binge-eat events in each study phase, were assessed by the Wilcoxon Rank Sum statistic. For CGIS and YBOCS scales, the Wilcoxon Rank Sum statistic was used to test the change from baseline for each subject, for every week of the study. Statistical significance was determined if a p-value was less than 0.05.

## Results

### Part I

#### PK evaluation (see Additional file 1)

A linear dose response relationship was demonstrated between Cohorts #1 (30 mg) and #2 (60 mg), with a factor of two between cohorts. By 90 min post-administration, Cohorts #1 and #2 achieved an average topiramate plasma concentration of 0.16 µg/ml and 0.3 µg/ml, respectively. These plasma levels were maintained up to 540 min after administration. In Cohort #3 concentration levels demonstrated a cumulative dose response relationship with an additive increase in concentrations, and achieved a peak average level of 2 µg/ml. By 24 h plasma levels showed a decline in all three cohorts, though remained near peak levels (Graphic PK depiction in Additional file 1: Fig. S2a, b).

#### Adverse events and side effects

There were no adverse events during all 48 administrations in Part 1. There were no significant changes in clinical laboratory tests following SipNose-topiramate administration. General physical and nasal mucosa examinations were normal in all subjects in all cohorts.

One side effect was reported in Cohort #1: sore throat (12.5%), which is a known topiramate related side effects and doesn’t necessarily result from the combination product.

Two side effects were reported in Cohort #2: headache (12.5%) and runny nose (12.5%).

Eleven side effects were reported in Cohort #3, all of which appeared after the highest dose (180 mg) was given: dizziness (9.09%), heaviness (4.54%), mood changes (4.54%), tiredness (4.54%), headache (4.54%), flu-like symptoms (4.54%), impaired ability to concentrate (4.54%), blurred vision (4.54%), diarrhea (4.54%), lack of appetite (4.54%),

### Part II

The number of individual binge-eating or urge to binge-eat events, as well as the number of days in which binge-eating or urge to binge-eat events occurred were collected from patient diaries. Table 2 tally of binge-eating events and urges to binge-eat events presents the number of binge-eating events, urges to binge-eat, mean number of binge-eating events per week, mean number of binge eating event days per week, mean number of urges to binge-eat per week and mean number of urge to binge-eat days per week. During the three phases there were a total of 135 binge-eating events and 71 urge to binge-eat events in the two-week baseline phase, 199 binge-eating events and 295 urge to binge-eat events in the 8-week treatment phase, and 64 binge-eating events and 27 urge to binge-eat events in the two-week follow-up phase.

**Table 2:** Tally of binge-eating events and urges to binge-eat events

	Total # binges to eat	Total # urges to binge-eat	Number of binges per week Mean (± SD)	Difference from baseline in # binges-eating eventst per week Range (p-value)	Number of binge-eating event days per week Mean (± SD)	Difference from baseline in # binge-eating event days per week Range (p-value)	Number of Urges to Binge-eat Per Week Mean (± SD)	Difference from baseline in # Urge-to-binge-eat per week Range (p-value)	Number of urges to binge-eat days per week Mean (± SD)	Difference from baseline in # urges to binge-eat days per week Range (p-value)
Baseline phase	135	71	4.9 (± 2.26)		4.1 (± 1.49)		2.56 (± 1.66)		2.12 (± 1.33)	
Treatment phase	199	295	1.58 (± 2.23) to 2.67 (± 3.63)	− 2.3 to − 3.4 (p-value 0.0005–0.0034)	1.32 (± 2.03) to 2.08 (± 2.27)	− 2.8 to − 2.0 (p-value 0.0005–0.005)	2.20 (± 2.62) to 4.67 (± 3.37)	-0.4 to 2.11 (p-value 0.0176–1)	1.67 (± 1.72) to 3.25 (± 2.01)	− 0.2 to 1.13 (p-value 0.0420–0.6377)
Follow-up phase	64	27	2.67 (± 3.09)	− 2.3 (± 2.35)	2.24 (± 2.20)	− 1.8 (± 1.90)	1.15 (± 1.41)	− 1.4 (± 1.41)	2.24 (± 2.20)	− 1.1 (± 1.09)

Number of binge-eating events, urges to binge-eat and mean number of binge-eating events per week, mean number of binge-eating event days per week, mean number of urges to binge-eat per week and mean number of urge to binge-eat days per week, respectively

Participant reported that 66% of urge to binge-eat events in the treatment phase (196) were treated (some after beginning to binge-eat) of which 81.1% (159) were treated with 60 mg (one dose), 16.3% (32) with 120 mg (two doses), and 2.6% (5) with 180 mg (three doses).

In 86% of treated urge to binge-eat events (169 of 196), patients reported that treatment was helpful.

*Mean weekly number of binge-eating events* Composite means are presented in Additional file 1: Table S1. The mean number of binge-eating events per week during the baseline phase was 4.9 (± 2.26). During the 8-week treatment phase the number of binge-eating events per week decreased and ranged from 1.58 (± 2.23) to 2.67 (± 3.63) at the end of the treatment phase. The mean number of binge-eating events during the two-week follow-up phase was 2.67 (± 3.09).

Statistically significant differences from baseline period to treatment period were found at each treatment week, with the decrease in mean number of binge-eating events ranging from − 2.3 (± 1.89) to − 3.4 (± 1.70) per week (p-values ranging from 0.0005 to 0.0034).

The overall mean number of binge-eating events per week for the treatment phase also differed significantly from baseline (p = 0.0005).

The mean number of binge-eating events per week in the follow-up phase wasn’t statistically significantly different from treatment phase mean (p = 0.2881) but did differ significantly from the baseline phase (p = 0.0210).

*Mean number of binge-eating event days per week* The mean number of binge-eating event days per week during the baseline phase was 4.08 (± 1.49). The mean number of binge-eating event days per week during the treatment phase decreased and ranged from 1.32 (± 2.03) to 2.08 (± 2.27). The mean number of binge-eating event days during the follow-up phase was 2.24 (± 2.20).

Statistically significant differences from baseline to treatment phase were found at each week, with decrease in binge-eating events ranging from -2 to -2.8 (p-values ranging from 0.0005 to 0.0049). Follow-up phase mean number of binge-eating event days was not statistically significant different from treatment period (p = 0.2246).

*Mean weekly number of urge to binge-eat events* The mean number of urges to binge-eat events per week during the baseline phase was 2.56 (± 1.66). Mean number of urges to binge-eat events per week during the treatment phase ranged from 4.67 (± 3.37) to 2.20 (± 2.62). The mean number of urges to binge-eat events per week during the follow-up phase was 1.15 (± 1.41).

The difference between baseline and treatment phases was statistically insignificant (p = 0.8501). The mean number of urges to binge-eat events per week during the follow-up phase differed significantly from the treatment (p = 0.0029) and from the baseline phases (p = 0.0034).

*Mean number of urge to binge-eat event days per week* The mean number of urge to binge-eat event days per week during the baseline phase was 2.12 (± 1.33). The mean number of urge to binge-eat event days per week during the treatment phase ranged from 1.67(± 1.72) to 3.25(± 2.01). The mean number of urge to binge-eat event days per week during the follow-up phase was 0.98 (± 1.23).

The difference between baseline and treatment phases was statistically insignificant (p = 0.9697). The mean number of urge to binge-eat event days per week during the follow-up phase differed significantly from the treatment (p = 0.0059) and from the baseline phases (p = 0.0063).

**Fig. 2:**
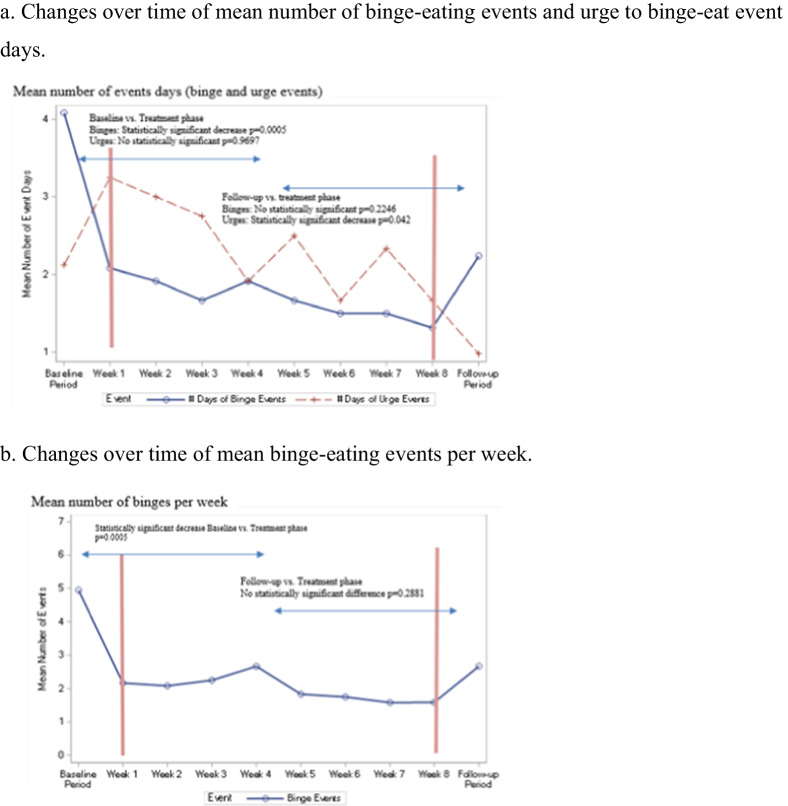
**a, b** Changes in variables between weeks. **a** Changes over time of mean number of binge-eating events and urge to binge-eat event days **b** Changes over time of mean binge-eating events per week

Changes in variables between weeks are presented graphically in Fig. 2a, b.

#### Patient severity of illness and post-treatment condition scoring

Significances of Patient Severity of Illness and Post-treatment Condition Scoring, between weeks of treatment can be seen in Additional file 1: Table S2.

CGI-S: There was a statistically significant decrease during all 8 weeks of the treatment phase when compared with the baseline phase (p = 0.002–p = 0.0156).

*YBOCS-BE* There was a statistically significant decrease during all 8 weeks of the treatment phase when compared with the baseline phase (p = 0.002–p = 0.0161).

#### Adverse events and side effects

There were no adverse events reported during all 251 treatments in the treatment phase.

Safety monitoring revealed no deviation from baseline for all parameters: physical examination (including an ear, nose and throat (ENT) exam); vital signs (blood pressure, pulse rate, and temperature); blood laboratory tests (biochemistry and CBC), and urine toxicology (cannabinoids screen, benzodiazepine screen, amphetamine screen, methadone metabolite).

The following side effects were reported during the 8 weeks of treatment: headache (5.17%), sneezing (4.38%), tiredness (1.59%), and nausea (3.98%). Affected patients did not consider these as serious, no medical treatment was needed, and no alteration of treatment was needed as a result.

Some patients reported a lingering bitter taste in the naso/oropharynx after administering treatment doses.

## Discussion

This study presents a novel “as needed” approach to binge-eating management by combining topiramate’s potential as binge eating disorder pharmacotherapy with intranasal direct nose to brain drug delivery. Several CNS pharmacotherapies with good BBB penetration were recently approved for intranasal administration via intranasal delivery products that deliver drugs to the systemic circulation. These include S-ketamine for the treatment of major depression [32], midazolam in patients with epilepsy 12 years of age and older [45], sumatriptan nasal powder for acute migraine headache treatment [46], and naloxone for opioid overdose [47].

We selected the SipNose product due to its unique approach to intranasal drug delivery. The SipNose product differs from existing methods since it takes advantage of the nasal cavity's physio-anatomy that allows efficient drug absorption and delivery directly to the brain [35]. Existing, commercially available intranasal drug-delivery devices deliver aerosol mainly to the lower and mid-nasal cavity turbinates and only minor amounts if any to the upper nasal cavity turbinates, thus allowing systemic delivery via the vascular rich mucosa in the middle-turbinate region. In contrast, SipNose technology delivers higher aerosol percentages to the upper nasal cavity, thus allowing limited systemic circulation delivery and pronounced direct delivery from the olfactory epithelium to the brain. SipNose’s innovative approach enables broad, consistent drug delivery to the upper area of the nasal cavity, which improves active drug delivery to the brain when compared with extant commercial nasal delivery devices. The platform offers an alternative to traditional nasal inhalers, as well as to tablets and injections, mainly in the field of central nervous system (CNS) pharmacotherapy.

In the study's first part, topiramate's pharmacokinetic profile for SipNose intranasal delivery demonstrated a linear dose response relationship between Cohorts 1 and 2 and a cumulative increase in drug plasma concentrations in Cohort 3. The linear dose response is consistent with the linear dose response of oral topiramate. The additive concentration increases in Cohort 3 was also consistent with known data regarding the drug's long t_1/2_ [48]. Part I results preliminarily demonstrate SipNose’s ability to deliver topiramate dry formulation in a consistent and controlled fashion. It also demonstrates the nasal mucosa’s ability to provide consistent and controlled topiramate absorption when applied with the SipNose device, even when administered as consecutive doses. When comparing with oral dosing, Doose et al. demonstrated maximum plasma concentrations of 1.73–28.7 μg/mL at oral doses of 100–1200 mg, respectively. In part I PK data demonstrated plasma concentrations of 0.16–2 μg/mL at IN doses of 60–180 mg. Part II proof-of-concept results, point towards the potential clinical efficacy of IN PRN topiramate administration using the lower dosing ranges tested in part I. The combination of Parts I and II results, therefore, indicate that IN topiramate may be efficacious at lower doses and with lower drug exposure. This is further supported when comparing Part II results with oral dosing. The required daily dose of oral topiramate in daily usage BED treatment is ~ 180 mg or more (≥ 1260 mg/week) [22–24]. In contrast, we estimate that study patients were exposed to an average of 114–193 mg/week (1.58–2.67 binge-eating events/week, 81% treated with 60 mg, 16% with 120 mg and 2.5% with 180 mg). This amounts to a 6.5–11-fold reduction in drug exposure as compared with the daily oral topiramate usage. However, the study’s small sample size limits these preliminary conclusions and larger sampling is needed for further validation. Furthermore, the PK data in Part I may not be generalizable to the BED patient population since Part I PK was assessed in individuals with lower BMI’s than the patients in Part II. This was intentional, as Part I data focused on preliminary PK evaluation in healthy individuals, and Part II on BED patients who often suffer from obesity as a direct result of the BED. None the less, larger PK studies are needed to verify baseline PK data in healthy and overweight individuals.

The SipNose-topiramate’s success in reducing binge-eating event numbers in Part II is proof-of-concept for its ability to rapidly reach and effect the CNS, long before it reaches peak levels (around 90 min). To that end, the direct nose-to-brain delivery offers a pharmaco-distribution and kinetic profile that is the reverse of, and better than, systemic dosing. Systemic dosing requires drug delivery to plasma at levels sufficiently elevated to allow for crossing the BBB. In contrast, DNTB delivery allows for rapid near-direct CNS drug delivery with subsequent plasma distribution. As such, any rapid rise in plasma levels demonstrated by DNTB delivery likely underestimates the rapid rate of rise of CNS tissue topiramate levels. Additionally, lower DNTB plasma levels may allow for an improved systemic side effect profile. Further study is needed to determine these assumptions. Such study should include a larger patient cohort, longer treatment period and a comparison group treated daily with oral topiramate.

This study’s second part aimed to demonstrate SipNose-topiramate’s clinical proof of concept of its potential efficacy as an acute “as needed” treatment for reducing binge-eating event frequency and illness severity. To our knowledge, this is the first study that utilizes topiramate for acute PRN binge-eating event treatment rather than preventative daily therapy. Study results demonstrated that this method was well received by patients who felt that the treatment was effective in helping them control their urges to binge-eat and prevent binge-eating event progression. This is supported by the reduction in number of binge-eating events and binge-eating event days per week in the treatment phase, as well as by the reduction in CGI-S and YBOCS-BE disease severity scales. The significant reduction in both scores during all treatment weeks when compared to baseline period, is consistent with the reduction in number of binge-eating events during the treatment phase. We further introduce the concept of monitoring urges to binge-eat as a point for PRN treatment. The lack of urge to binge-eat reduction during the treatment phase is consistent with author anticipation of IN topiramate as a short-acting acute treatment in reducing binge-eating event severity and/or allowing the patient better cognitive control in overcoming urges to binge-eat.

Interestingly, the number of binge-eating events remained reduced in the follow-up phase. This “tail effect” was one of the study’s secondary outcomes. We hypothesize that the reduction in binge-eating events after the end of the drug treatment is a long-term behavioral effect induced by self-treatment. We suggest that during the treatment phase, patients had to engage in a mindful cognitive pause to assess urge to binge-eat severity and decide whether it required treatment. This may have positively influenced obsessive thinking. This reflective behavior pattern may have remained and improved patients' self-control and self-esteem. It may also explain another secondary outcome, the reduction in number of urges to binge-eat events during the follow-up phase. The new behavior pattern may have allowed patients the ability to redefine their urge to binge-eat severity. During the follow up phase, patients may have downgraded their urge to binge-eat severity assessment such that they completely dismissed or disregarded urges to binge-eat of lower intensity which they would have previously considered significant. Further research is needed with longer treatment and follow-up phases, including follow-up YBOCS-BE monitoring to assess this phenomenon.

In terms of drug safety as a primary outcome, the lack of adverse events in both Parts I and II suggests that SipNose-topiramate nasal delivery is safe for clinical use in doses ranging from 60 mg and up to 180 mg per day. Most side effects were reported in Part I Cohort #3, in which a high dose of topiramate was taken (180 mg). In the second part, this constituted less than 3% of self-administered doses. A number of these side effects (headache, sneezing, tiredness and nausea) were also reported by patients in Part II, though it is unknown whether these were associated with higher doses. Furthermore, all reported side effects were low in frequency in both study parts, did not pose a health hazard to study participants, and did not require treatment. All are also known topiramate related side effects [22–24, 49] and don’t seem to result from the combination product. The only effect that may to be related to the combination product was the bitter taste reported by patients in Part II, though taste perversion due to oral topiramate has been reported previously [24]. In retrospect, study medical staff viewed the lingering bitter taste as a potentially advantageous. Firstly, it provides sensory confirmation of medication delivery, like the sensation of a pill entering the stomach. Secondly, it may prevent over/unnecessary dosing and addiction.

The low side effect frequency highlights the potential advantage of using the SipNose-topiramate product, in which more than 97% of individual effective doses were relatively low (60 or 120 mg), as was the cumulative dose per week. This contrast sharply with a higher side effect and adverse event frequency associated with oral topiramate at higher doses [22–24, 49] including one study that reported a patient dropout rate of ~ 32% [49] (14 of 44 patients) due to adverse events. Additionally, the intermittent “as needed” dosing allowed for an average greater time lapse and drug clearance between treatment days, thereby reducing daily usage steady state drug exposure. The reduced number of urges to binge-eat in the follow-up phase suggest that a longer treatment phase could have resulted in even further drug exposure reduction through a gradual reduction in urges to binge-eat. Further research with a longer treatment period is necessary to confirm this.

The study has several limitations. Firstly, the study is designed as preliminary “proof-of-concept.” Both parts included a small number of volunteers and patients, respectively. Secondly, the study group in Part II was not compared to a control rather to their own measurements during the baseline period, prior to commencing therapy. Prior studies have demonstrated a strong placebo effect in BED treatment [50, 51]. The lack of a control group limits the study's ability to distinguish between true treatment and placebo effect. Larger, randomized-control groups with longer treatment and follow-up phases may yield more accurate and robust results. Thirdly, the 8-week treatment and 2-week follow-up phases in Part 2 may have been too brief to conclusively determine potential long term treatment effect. In addition, due to the short study time period, data was not collected about participants who became completely abstinent. Also, the CGI-S and YBOCS-BE were not evaluated during the follow-up phase. Extending their evaluation in the follow-up phase can lend broader and deeper understanding of BED's cognitive and behavioral components with an IN PRN treatment method.

Our study suggests several potential advances in the BED therapeutic field. We introduce as proof-of-concept the first acute, as-needed, direct nose-to-brain intranasal therapy for a psychiatric illness, with reduced drug exposure and side effect profile. Specifically, in this study, the SipNose-topiramate direct nose-to-brain drug delivery combination provided safe, as-needed treatment for patients suffering from binge-eating disorder and succeeded in reducing disease severity. Study findings point towards positive efficacy though this is limited by the study’s preliminary design with a small sample size. Additionally, BED is currently defined solely based on the number and frequency of binge-eating events without accounting for urges to binge-eat events as a treatment requisite. The introduction of as-needed pharmaco-therapy highlights the importance of considering urges to binge-eat and their subjective intensity as a as a potential preliminary point for therapeutic action.

In conclusion, we present a two-part study that introduces the use of the SipNose-topiramate device-drug combination as a potential safe and effective acute “as-needed” therapy for binge eating disorder. Study results reveal a potential new therapy method for reducing binge-eating events in BED patients, that is well received by patients, has a preliminarily predictable PK and drug safety profile, with a positive proof of concept of usability and feasibility, and that can potentially improve patient's health and quality of life. The study is limited by its small sample size and open label design with no placebo arm and relatively short treatment and follow-up phases. None the less, this preliminary study demonstrates the therapeutic potential of the SipNose-topiramate combination product. Further research is needed to validate these results and elucidate the treatments' long-term efficacy and influence on BED therapy.

## Supplementary Information

**Additional file 1**: Supplementary Data.** Figure S1.** Clinical evidence for SipNose superior delivery to the olfactory epithelium.** Figure S2.** Cohorts 1, 2 and 3 – Average topiramate plasma concentrations vs. time.** Table S1.** Changes in variables between phases.** Table S2.** Significances of patient severity of illness and post-treatment condition scoring, between weeks of treatment.

## Data Availability

Not applicable.
