# Supplementary material for: SipNose-topiramate: a potential novel approach to binge eating management
Source: J Eat Disord. 2023 Jun 26;11:102. doi: 10.1186/s40337-023-00825-9 (PMC10294342; doi:10.1186/s40337-023-00825-9)
Supplement: Supplementary file 1 — Additional file 1: Supplementary Data. Figure S1. Clinical evidence for SipNose superior delivery to the olfactory epithelium. Figure S2. Cohorts 1, 2 and 3 – Average topiramate plasma concentrations vs. time. Table S1. Changes in variables between phases. Table S2. Significances of patient severity of illness and post-treatment condition scoring, between weeks of treatment. [file 40337_2023_825_MOESM1_ESM.docx]

**SUPPLEMENTARY DATA**

**Supplementary Figures**

**Figure S1: Clinical evidence for SipNose superior delivery to the olfactory epithelium**


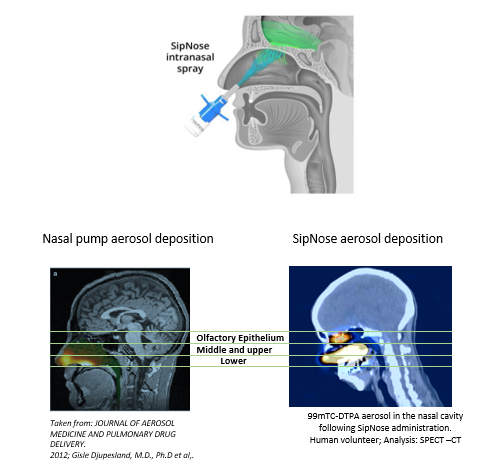


**Fig. S1 a,b** Comparison between localization of aerosolized drug in the nasal cavity, following the use of a commercial nasal pump delivery (**left**) and the use of the SipNose device delivery (**right**).

**[Figure S2: Cohorts 1, 2 and 3 – Average topiramate plasma concentrations vs. time](#FigureS2intext)**

**a.**


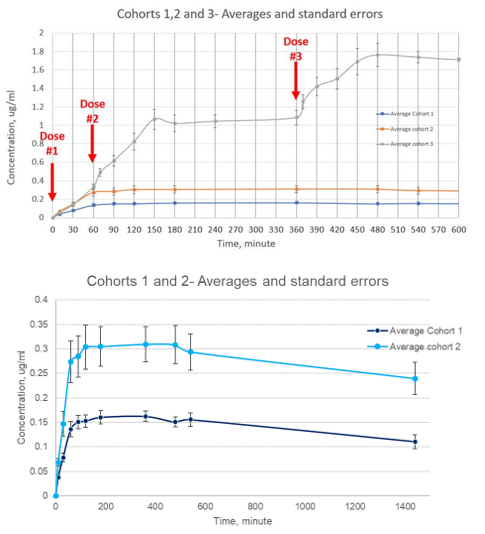


**b.**


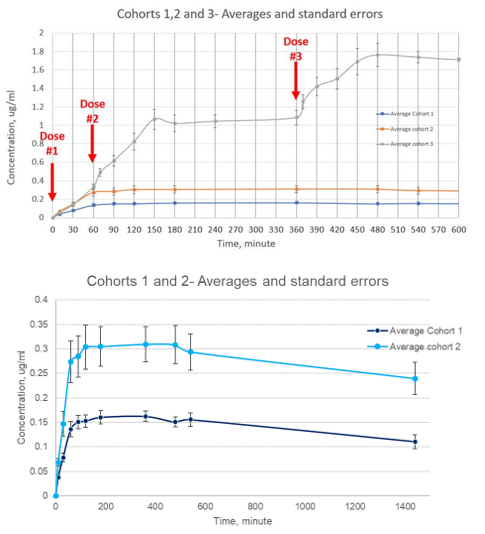


**Fig. S2 a,b** Plasma concentrations Vs. time in Part I -pharmacokinetics (PK) study. **a** topiramate’s pharmacokinetic profile for SipNose intranasal delivery for cohorts 1,2 (single administration) and cohort 3 (3 sequential administrations). **b** Topiramate’s pharmacokinetic profile for cohorts 1 and 2.

**Supplementary Tables:**

**[Table S1](#TableS2intext)** [Changes in variables between phases](#TableS2intext)

| Variable | Baseline vs. treatment periods | Follow-up vs. treatment periods | Follow-up vs. baseline periods |
| --- | --- | --- | --- |
| Mean number of binges to eat per week | **p=0.0005** | p=0.2881 | **p=0.0212** |
| Mean number of binge-eating event days per week | **p=0.0005** | p=0.2246 | **p=0.0098** |
| Mean number of urges to binge-eat per week | p=0.8501 | **p=0.0029** | **p=0.0034.** |
| Mean number of urge to binge- eat event days per week | P=0.9697 | **p= 0.0059** | **p= 0.0063** |

**Table S1** Statistical analysis of changes in mean number of binges to eat per week, binge-eating event days per week, urges to binge-eat per week and urge to binge- eat event days per week between the three different study periods (baseline, treatment and follow-up).

Statistically significant values (p<0.05) appear in bold.

**[Table S2](#TableS3intext)** [Significances of patient severity of illness and post-treatment condition scoring, between weeks of treatment.](#TableS3intext)

|  | CGI-S | YBOCS-BE (Total) |
| --- | --- | --- |
| Baseline vs. Week 1 | **p=0.0156** | **p=0.0020** |
| Baseline vs. Week 2 | **p=0.0107** | **p=0.0107** |
| Baseline vs. Week 3 | **p=0.0078** | **p=0.0020** |
| Baseline vs. Week 4 | **p=0.0039** | **p=0.0010** |
| Baseline vs. Week 5 | **p=0.0020** | **p=0.0078** |
| Baseline vs. Week 6 | **p=0.0020** | **p=0.0098** |
| Baseline vs. Week 7 | **p=0.0078** | **p=0.0161** |
| Baseline vs. Week 8 | **p=0.0020** | **p=0.0063** |

**Table S2** Statistical analysis of changes in CGI-S and YBOCS-BE scoring between the baseline week and each of the treatment weeks (week 1 to week 8).

Statistically significant changes (p<0.05) appear in bold.
